# Supplementary material for: Comparison of Ameliorative Effects between Probiotic and Biodegradable Bacillus subtilis on Zearalenone Toxicosis in Gilts
Source: Toxins (Basel). 2021 Dec 10;13(12):882. doi: 10.3390/toxins13120882 (PMC8703852; doi:10.3390/toxins13120882)
Supplement: Supplementary file 1 [file toxins-13-00882-s001.zip › toxins-1489363-supplementary.pdf]

Supplementary Materials: Comparison of Ameliorative Effects between Probiotic and Biodegradable *Bacillus subtilis* on Zearalenone Toxicosis in Gilts

Wenqiang Shen, Yaojun Liu, Xinyue Zhang, Xiong Zhang, Xiaoping Rong, Lihong Zhao, Cheng Ji, Yuanpei Lei, Fengjuan Li, Jing Chen and Qiugang Ma

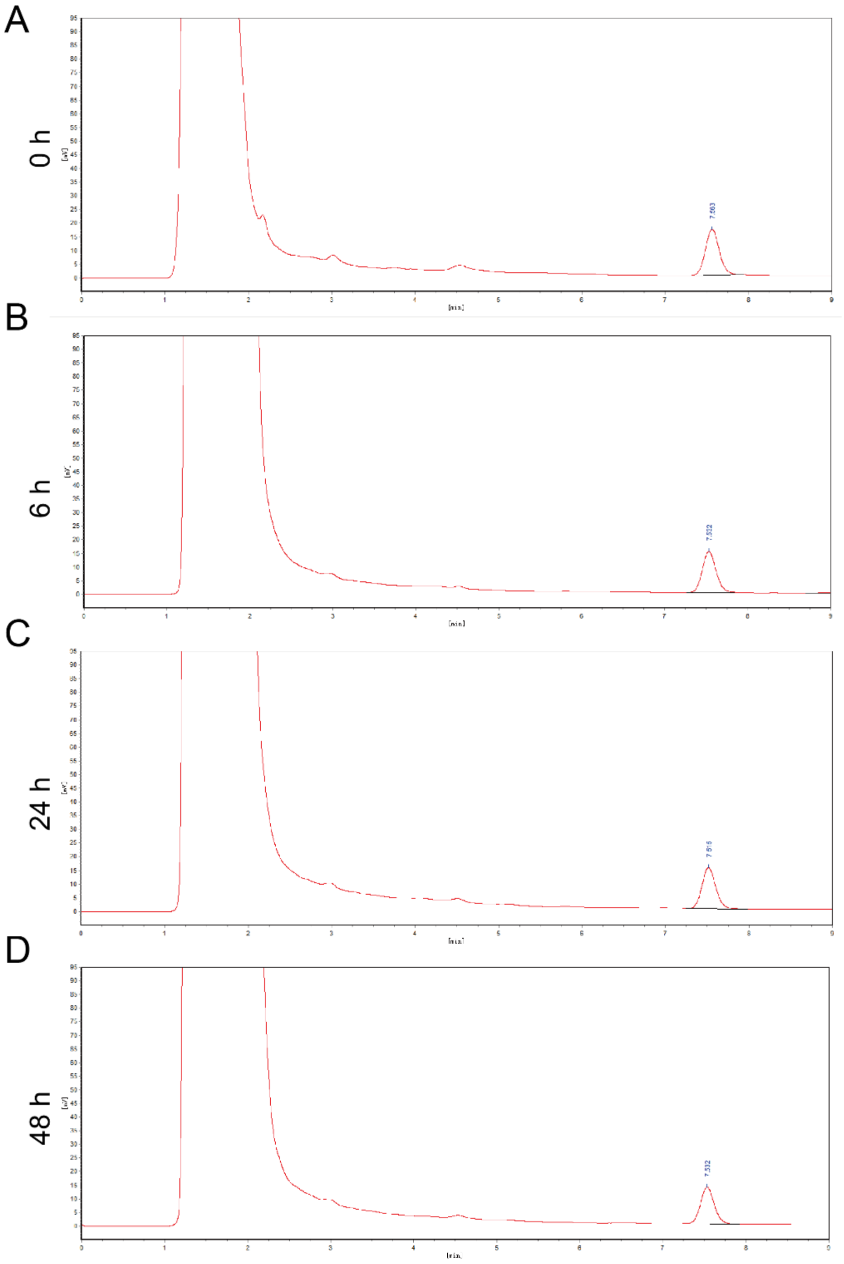

Figure S1. The representative HPLC-chromatograms of ZEN-degradation by *Bacillus subtilis* ANSB010 at 0h (A), 6h (B), 24h(C) and 48h (D). x-axis, retention time (min); y-axis, response value (mAU).

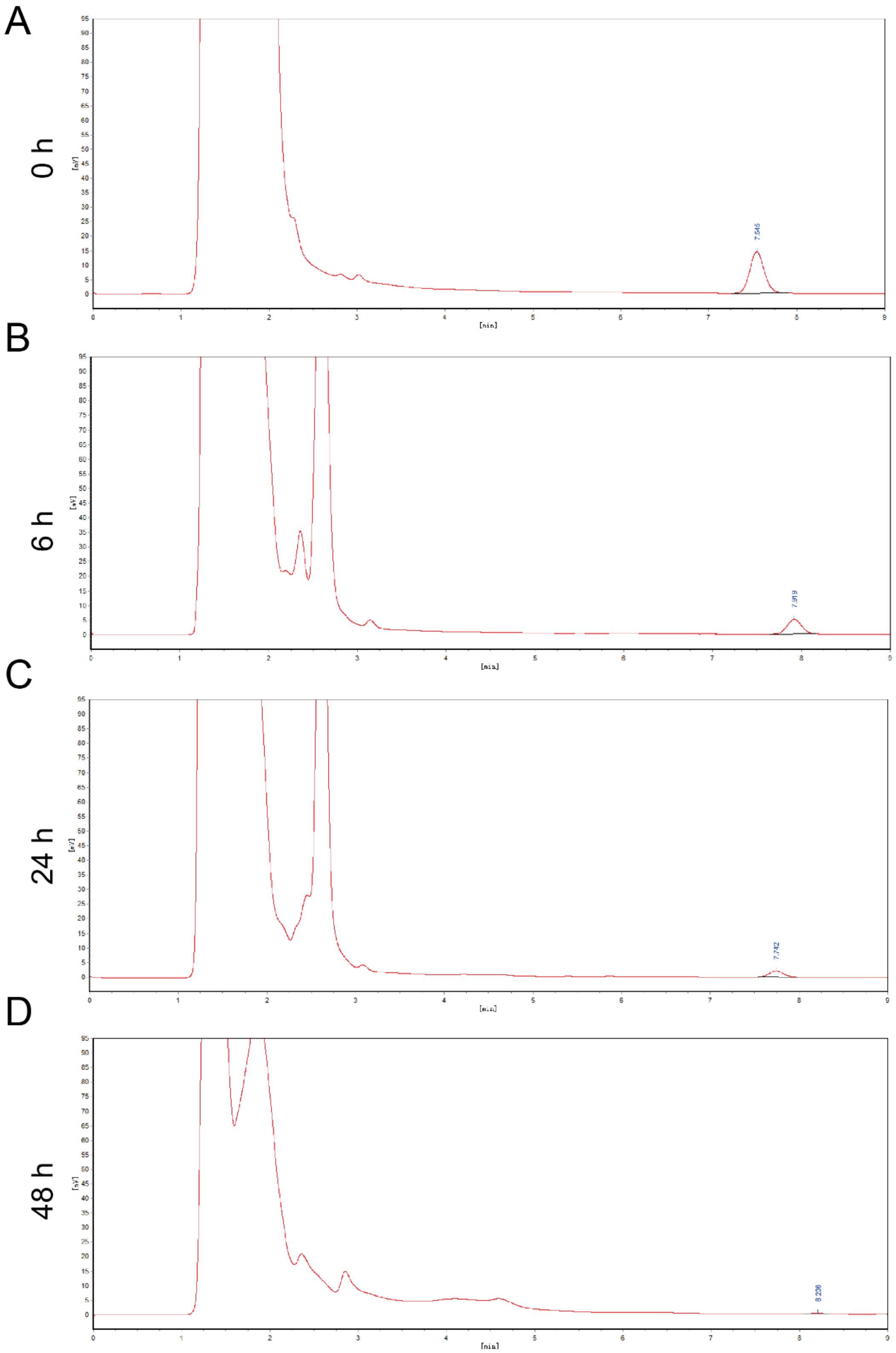

Figure S2. The representative HPLC-chromatograms of ZEN-degradation by *Bacillus subtilis* ANSB01G at 0h (A), 6h (B), 24h (C) and 48h (D). x-axis, retention time (min); y-axis, response value (mAU).

16S rDNA sequence of ANSB010:

GCTCCCTGATGTTAGCGGCGGACGGGTGAGTAACACGTGGGTAACCTGCCTGTAA
GACTGGGATAACTCCGGGAAACCGGGGCTAATACCGGATGGTTGTTTGAACCGCA
TGGTTCAAACATAAAAGGTGGCTTCGGCTACCACTTACAGATGGACCCGCGGCGC
ATTAGCTAGTTGGTGAGGTAACGGCTCACCAAGGCAACGATGCGTAGCCGACCTG
AGAGGGTGATCGGCCACACTGGGACTGAGACACGGCCCAGACTCCTACGGGAGG
CAGCAGTAGGGAATCTTCCGCAATGGACGAAAGTCTGACGGAGCAACGCCGCGT
GAGTGATGAAGGTTTTCGGATCGTAAAGCTCTGTTGTTAGGGAAGAACAAGTACC
GTTCGAATAGGGCGGTACCTTGACGGTACCTAACCAGAAAGCCACGGCTAACTAC
GTGCCAGCAGCCGCGGTAATACGTAGGTGGCAAGCGTTGTCCGGAATTATTGGGC
GTAAAGGGCTCGCAGGCGGTTTCTTAAGTCTGATGTGAAAGCCCCCGGCTCAACC
GGGGAGGGTCATTGGAAACTGGGGAACTTGAGTGCAGAAGAGGAGAGTGGAATT
CCACGTGTAGCGGTGAAATGCGTAGAGATGTGGAGGAACACCAGTGGCGAAGGC
GACTCTCTGGTCTGTAACTGACGCTGAGGAGCGAAAGCGTGGGGAGCGAACAGG
ATTAGATACCCTGGTAGTCCACGCCGTAAACGATGAGTGCTAAGTGTTAGGGGGTT
TCCGCCCCTTAGTGCTGCAGCTAACGCATTAAGCACTCCGCCTGGGGAGTACGGTC
GCAAGACTGAAACTCAAAGGAATTGACGGGGGCCCGCACAAGCGGTGGAGCATG
TGGTTTAATTCGAAGCAACGCGAAGAACCTTACCAGGTCTTGACATCCTCTGACAA
TCCTAGAGATAGGACGTCCCCTTCGGGGGCAGAGTGACAGGTGGTGCATGGTTGT
CGTCAGCTCGTGTCGTGAGATGTTGGGTTAAGTCCCGCAACGAGCGCAACCCTTGA
TCTTAGTTGCCAGCATTCAGTTGGGCACTCTAAGGTGACTGCCGGTGACAAACCGG
AGGAAGGTGGGGATGACGTCAAATCATCATGCCCCTTATGACCTGGGCTACACAC
GTGCTACAATGGACAGAACAAAGGGCAGCGAAACCGCGAGGTTAAGCCAATCCC
ACAAATCTGTTCTCAGTTCGGATCGCAGTCTGCAACTCGACTGCGTGAAGCTGGAA
TCGCTAGTAATCGCGGATCAGCATGCCGCGGTGAATACGTTCCCGGGCCTTGTACA
CACCGCCCGTCACACCACGAGAGTTTGTAACACCCGAAGTC

16S rDNA sequence of ANSB01G:

TGCAGTCGAGCGGACAGATGGGAGCTTGCTCCCTGATGTTAGCGGCGGACGGGTG
AGTAACACGTGGGTAACCTGCCTGTAAGACTGGGATAACTCCGGGAAACCGGGGC
TAATACCGGATGGTTGTTTGAACCGCATGGTTCAGACATAAAAGGTGGCTTCGGCT
ACCACTTACAGATGGACCCGCGGCGCATTAGCTAGTTGGTGAGGTAACGGCTCAC
CAAGGCGACGATGCGTAGCCGACCTGAGAGGGTGATCGGCCACACTGGGACTGA
GACACGGCCCAGACTCCTACGGGAGGCAGCAGTAGGGAATCTTCCGCAATGGAC
GAAAGTCTGACGGAGCAACGCCGCGTGAGTGATGAAGGTTTTCGGATCGTAAAGC
TCTGTTGTTAGGGAAGAACAAGTGCCGTTCAAATAGGGCGGCACCTTGACGGTAC
CTAACCAGAAAGCCACGGCTAACTACGTGCCAGCAGCCGCGGTAATACGTAGGTG
GCAAGCGTTGTCCGGAATTATTGGGCGTAAAGGGCTCGCAGGCGGTTTCTTAAGTC
TGATGTGAAAGCCCCCGGCTCAACCGGGGAGGGTCATTGGAAACTGGGGAACTTG
AGTGCAGAAGAGGAGAGTGGAATTCCACGTGTAGCGGTGAAATGCGTAGAGATG
T6GGAGGAACACCAGTGGCGAAGGCGACTCTCTGGTCTGTAACTGACGCTGAGGA
GCGAAAGCGTGGGGAGCGAACAGGATTAGATACCCTGGTAGTCCACGCCGTAAA
CGATGAGTGCTAA7GTGTTAGGGGGTTTCCGCCCCTTAGTGCTGCAGCTAACGCAT

TAAGCACTCCGCCTGGGGAGTACGGTCGCAAGACTGAAACTCAAAGGAATTGACG
GGGGCCCGCACAAGCGGTGGAGCATGTGGTTTAATTCGAAGCAACGCGAAGAAC
CTTACCAGGTCTTGACATCCTCTGACAATCCTAGAGATAGGACGTCCCCTTCGGGG
GCAGAGTGACAGGTGGTGCATGGTTGTCGTCAGCTCGTGTCGTGAGATGTTGGGTT
AAGTCCCGCAACGAGCGCAACCCTTGATCTTAGTTGCCAGCATTCAGTTGGGCACT
CTAAGGTGACTGCCGGTGACAAACCGGAGGAAGGTGGGGATGACGTCAAATCAT
CATGCCCCTTATGACCTGGGCTACACACGTGCTACAATGGACAGAACAAAGGGCA
GCGAAACCGCGAGGTTAAGCCAATCCCACAAATCTGTTCTCAGTTCGGATCGCAG
TCTGCAACTCGACTGCGTGAAGCTGGAATCGCTAGTAATCGCGGATCAGCATGCC
GCGGTGAATACGTTCCCGGGCCTTGTACACACCGCCCGTCACACCACGAGAGTTTG
TAACACCCGAAGTCGG TGAGGTAACCTTTAGGAGCC AGCCGCCGAAGG
